# Music Therapy During COVID-19: Changes to the Practice, Use of Technology, and What to Carry Forward in the Future

**DOI:** 10.3389/fpsyg.2021.647790

**Published:** 2021-05-21

**Authors:** Kat R. Agres, Katrien Foubert, Siddarth Sridhar

**Affiliations:** ^1^Yong Siew Toh Conservatory of Music, National University of Singapore, Singapore, Singapore; ^2^LUCA School of Arts, Leuven, Belgium; ^3^Faculty of Biomedical Sciences, Department of Development and Regeneration, KU Leuven, Leuven, Belgium; ^4^University Psychiatric Centre, Kortenberg, Belgium; ^5^United World College of South East Asia, Singapore, Singapore

**Keywords:** music therapy, music technology, COVID-19 pandemic, online tools, teletherapy, regional practices, regional comparisons

## Abstract

In recent years, the field of music therapy (MT) has increasingly embraced the use of technology for conducting therapy sessions and enhancing patient outcomes. Amidst a worldwide pandemic, we sought to examine whether this is now true to an even greater extent, as many music therapists have had to approach and conduct their work differently. The purpose of this survey study is to observe trends in how music therapists from different regions around the world have had to alter their practice, especially in relation to their use of technology during the COVID-19 pandemic, because of limited options to conduct in-person therapy due to social distancing measures. Further, the findings aim to clarify music therapists’ perspectives on the benefits and limitations of technology in MT, as well as online MT. In addition, this survey investigated what changes have been necessary to administer MT during COVID-19, in terms of virtual therapy and online tools, and how the changes made now may affect MT in the future. We also explored music therapists’ views on whether special technology-focused training might be helpful to support the practice of MT in the future. This is the first survey, to our knowledge, to break down opinions of and trends in technology use based on geographical region (North America, Europe, and Asia), and several noteworthy differences were apparent across regions. We hope our findings provide useful information, guidance, and a global reference point for music therapists on effectively continuing the practice of MT during times of crisis, and can encourage reflection and improvement in administering MT.

## Introduction

### Music Therapy and Technology

Within the last 20 years, music therapy (MT) has embraced various technologies to try to improve the practice or expand the realm of possibilities offered to patients. Technology may be used to cater to the multifaceted needs of patients, be it in terms of patient capability, or accessibility and portability of digital tools. The primary goal of MT is to help patients achieve higher levels of wellbeing, using musical experiences and interaction with the therapist as dynamic forces of change (Kirkland, 2013; Bruscia, 2014). With this goal in mind, having a wider scope of digital MT tools available allows music therapists to select tools that will best suit their patients’ cognitive, emotional, and/or physical needs. It is widely accepted that utilizing new technology allows MT to become more inclusive to more individuals, often allowing physically limited patients to participate in MT that would not be possible using only traditional instruments (Magee, 2006; Hahna et al., 2012). This in turn can result in higher motivation levels to pursue and continue undergoing therapy. Technology also allows a greater degree of feedback to be generated while participating in MT, giving patients a greater sense of agency, participation, and purpose (Lem and Paine, 2011; Ramsey, 2011).

Therapists may be inclined to embrace certain methods and technologies depending on the diagnoses of their patients, as well as their approach to diagnostics (e.g., symptomatically, versus a more broad/holistic, multidimensional approach). Some considerations that may influence the use of technology are clinical contexts/applications, and the music therapist’s orientations (e.g., community music therapy, neurologic music therapy) and approaches (e.g., psychodynamic, humanistic, cognitive-behavioral, developmental), as well as factors external to the therapy or patient, such as accessibility and availability of funding. Another influence undoubtedly stems from the music therapist’s various beliefs and values regarding clinical practice and the therapeutic process, i.e., their own biases and cultural beliefs may influence how they approach working with patients. Finally, the therapists’ training, familiarity, and skills with regard to technology will also play a crucial role in the adoption of new technologies.

Although a full review of the technologies available for various clinical applications is not possible here, we will briefly describe several examples of music-based technologies and therapeutic tools that may be employed to help those with physical and cognitive impairments, mental disorders, and emotional/social disorders, as well as support wellbeing and help foster one’s sense of identity and purpose.

Technology provides many options to aid those with physical impairments during MT, as traditional musical instruments are often too complex to handle. Adaptive Use Musical Instruments (AUMI), for example, are a form of technology that involves the creation of music in non-conventional ways, such as using a camera to track the body/head movements to different points on a screen in order to create music (Oliveros et al., 2011). Another example is a Brain Computing Music Interface (BCMI) system, a kind of neurofeedback technology that involves mapping neural signals from the brain to a music creation/generation system, an applicable tool for those who cannot use their body to create music (Miranda et al., 2011). Serious games (i.e., games created primarily for education, healthcare, etc, and not for the sole purpose of entertainment) that involve movements in response to musical stimuli have also been developed to support patients’ physical abilities and motor control, as well as their motivation to undergo strengthening exercises (Agres and Herremans, 2017). In general, incorporating technology into MT for those who are physically restricted can maintain or improve a patient’s condition, as well as boost morale and wellbeing.

Music technology may also make MT more accessible to those with cognitive impairments, such as those with neurodevelopmental and neurocognitive disorders. Voice Output Communication Aids (VOCA), for example, assist those who have trouble communicating orally, and are controlled by a series of switches to output vocal commands (Magee et al., 2011). Serious games have also been developed to support cognitive function, such as memory performance (Agres et al., 2019). Broadly speaking, technology can facilitate accessibility and inclusivity across a range of cognitive abilities, and can be particularly helpful for those who have difficulty with verbal communication.

Music technology has been also used to assess mental conditions/states through, for example, acoustic analysis of improvizational music performance (Luck et al., 2006; Streeter et al., 2012; Snape, 2020). When patients, who may have a broad range of psychopathologies and severity profiles, create music, their performance contains information that relates to underlying affective and communicative processes that occur during music making. For example, in a study with Borderline Personality Disorder (BPD) patients—patients who have trouble with interpersonal relationships, emotional instability, and impulsive behavior—music information retrieval (MIR) techniques were used to analyze improvizations in which a therapist and patient play in tandem on a single piano (Foubert et al., 2017). This approach yielded clear disparities between BPD patients and the healthy control group, and affords a new way of assessing non-verbal interactions and cognitive processes.

As for enhancing social-emotional competence in MT, recently, MT has been aided by applications that facilitate patients’ development of psychological resources that support adaptive growth and wellbeing. Technology may be used as an additional tool to learn beneficial ways of using music for one’s health (by better understanding personal music listening behavior in daily life), and this skill transfers to general emotion regulation ability (Randall and Rickard, 2013; Gold et al., 2017). To enhance patient wellbeing, the use of technology allows stress to be reduced, as seen when engaging in musical games (Whitehead-Pleaux et al., 2011; de Witte et al., 2020). In addition, using technology that incorporates resonance frequency breathing during therapy sessions has been shown to deepen and support interpersonal and emotional processes naturally occurring during therapy, e.g., helping with the regulation of excessive arousal (Brabant et al., 2017). Recently, BCMI systems have also been developed to aid emotion self-regulation in listeners, by providing music and neurofeedback to help listeners identify and train their emotion states, as measured through EEG (Ramirez et al., 2015; Ehrlich et al., 2019).

Finally, adopting music technology allows identity to be explored: music from a patient’s culture or background is more widely available via music technology (Whitehead-Pleaux et al., 2011), and personally tailored music can strengthen the formation of identity through “peak experiences” that provide meaning, purpose, and significance to our lives (Ruud, 1997). Technology can also afford personal choice and ownership, which are connected to agency and belonging, allowing individuals to be an agentic actor of their personal health promotion (Saarikallio, 2017). For instance, in youth culture, technology opens new pathways to access music that does not belong to mainstream popular music, giving young individuals a voice to explore their position and identity in relation to ethnicity, gender, and class, anchoring a sense of belonging.

In summary, from streaming services and recording technologies to Serious Games and wearable devices, various kinds of technology have been incorporated into the clinical practice of MT in order to help patients achieve higher levels of wellbeing. Increasingly, technology has evolved to better meet patients’ cognitive, emotional, and/or physical needs, and can help foster the patient’s motivation, purpose, and sense of identity. Certain technologies, such as new digital musical interfaces and teleconferencing tools, have improved accessibility for some patients. In addition, advancements in technology have assisted therapists with patient diagnostics and assessment. Although there is an increasing trend in the use of various types of technology (see Magee, 2006; Hahna et al., 2012), this uptake of technology is not universal, and seems to be influenced in part by factors such as the cultural context, MT methods/approach, and of course the patients’ diagnoses.

### Online Music Therapy, and the Challenge of Administering MT During the COVID-19 Pandemic

The global COVID-19 pandemic has affected the way MT may be practiced. Countries around the world have gone into lockdown and enforced strict social distancing measures, in which regular interactions with members outside of one’s household or immediate family are disallowed. This is a disturbance in the way MT is normally practiced, as music therapists often depend on face-to-face interactions with patients to achieve optimal outcomes. In order to allow MT to continue during the pandemic, music therapists have had to embrace alternative methods of conducting sessions, such as teletherapy, or virtual music therapy (VMT). This refers to the administering of MT remotely using online conferencing tools, such as Zoom, Skype, etc., which have made themselves prevalent in allowing other required activities such as schooling and work to take place. The maintenance of MT sessions is of the utmost importance to patients, especially during such times of crisis, in which patients might experience heightened levels of stress due to a lack of future perspective and the unpredictability of this global pandemic (Mastnak, 2020).

Amidst the changes required due to social distancing measures, concerns have been voiced regarding the efficacy of online therapy, stating that there is limited access to affective embodiment in human interaction, which is considered to be one of music’s key strengths in relation to both internal experiences and interpersonal interactions in MT (Saarikallio, 2019). Patients have also shown concern about the privacy of the therapist’s space during online sessions, as they had no full view of the therapist’s room, which has stimulated anxiety and paranoid thoughts in some patients (Desmet, 2020). In addition, Desmet (2020) found that the patients’ own family members were sometimes in the same room, causing an unsafe environment for the patient.

Therapists (and patients) have spoken about “digital depression,” where the digital means of communication and of being present is experienced as more exhausting than sessions in physical contact with the therapist (Desmet, 2020). In MT, new learning processes often take place in an environment where the musical sounds of the therapist are attuned to or resonate with physical body gestures and facial expressions of the patient (Foubert et al., 2020); affective experiences are thus grounded/embedded in the body, having vitalizing importance. Research shows that such an exchange between humans has an emotional but also vital importance on biological functions of the human body (Desmet, 2020). It is theorized in this work that in online sessions, participants are frustrated because they yearn to experience immediate and direct affective embodied exchanges. While the *content* of what is said may be unaffected, the musical interaction is a complex, dynamic process that involves subtle exchanges. It will therefore be affected in online sessions by slight delays, which can be perceived as a passivity of the other, and which also contributes to the phenomenon of depression (Desmet, 2020).

On the contrary, past studies examining the efficacy of teletherapy claim that this method has the potential to be as effective as traditional, real-life therapy, provided the online tools are used correctly (Lightstone et al., 2015). Teletherapy, studied in many forms such as telerehabilitation, also tends to increase accessibility, especially to those from poorer economic backgrounds, geographically isolated individuals, or those who have other mobility challenges (Spooner et al., 2019). Attending teletherapy sessions may also be generally more convenient, and have reduced travel costs, compared with traditional in-person therapy (Latifi et al., 2021). It should be noted that not all patients will find online sessions to improve convenience or accessibility (i.e., those who do not have an internet connection at home, or who do not have the skills required to use the technology). Technology can therefore be an obstacle for some individuals. That said, for the significant number of patients for whom technology does improve accessibility, online therapy may serve as an adequate substitute for traditional in-person sessions. This may be especially true during difficult and unusual times (such as the COVID-19 situation), in which in-person MT is not always a possibility. Indeed, some practitioners have been honing the art of teletherapy. Knott and Block (2020), for example, offer a model for VMT that describes how to develop VMT that carefully considers the clinical goals, and ensures that the online format is as accessible, appropriate (taking into consideration the patient’s abilities), and effective as possible to meet the patient’s needs. Further, Molyneux et al. (2020) describe how continuing to deliver sessions virtually provided support, continuity, and an ongoing connection for people living with dementia and their companions who were isolated in their own homes due to the national lockdown in the United Kingdom.

### The Current Survey: Investigating Changes to Music Therapy and Technology Use During COVID-19

Previous survey studies have been conducted to investigate music therapists’ attitudes and practices involving technology (Magee, 2006; Hahna et al., 2012). These studies involved understanding how technologies were being used, the drawbacks and limitations of the technology, and music therapists’ own opinions on technology within MT. They also helped establish why music technology was not quickly/easily adopted in the practice of MT. The reasons primarily center around a lack of training (Magee, 2006), and trends in using music technology in MT across different demographics such as gender and age (Hahna et al., 2012). In this study, we aim to provide an updated account of technology in MT, particularly within the year 2020, to shed light on the impact that the COVID-19 pandemic has had on the field of MT and technology use within the field.

One area of focus here is to examine the practice of MT and technology use by *geographical region*, and how different clinical applications, as well as the use of technology, reflect the region in which MT is practiced. MT practices differ across geographical locations, a consequence of being embedded in different government systems, health care systems, education, training, and the way in which health is viewed within the country (Gadberry et al., 2015). The different approaches and practices—didactic, medical, healing, psychotherapeutic, recreational and ecological practices (Bruscia, 2014)—make the rules of transaction and interaction in MT, and the common basis upon which to establish any intervention, very difficult (Ruud, 2000). On top of that, the practice is subject to idiosyncrasies within each culture (Ruud, 2000). These cultural influences shape therapists’ views and beliefs on their practice, their use of technology, and have a large impact on the therapeutic interactions’ role and quality (Wheeler and Baker, 2010).

This study aims to (a) provide a current overview of the technology used within MT, as well as music therapists’ experiences and views regarding the use of technology in MT, (b) examine the impact of COVID-19 on the practice of MT and use of technology within MT, (c) identify trends in technology use in MT with regard to geographical location, and (d) investigate the reasons for not using technology within MT. To address these goals, certified music therapists in several countries around the world were invited to participate in a brief online survey containing questions about the music therapist’s background and practice, their experiences and views about conducting therapy using technology, and their experiences practicing MT during the COVID-19 pandemic. This study seeks to clarify how the pandemic has influenced the practice of MT in general, and how music therapists embrace technology in particular. Finally, by examining technology-related limitations (such as a lack of specialized training, and a lack of bespoke, accessible, human-centric technologies), this study aims to identify what changes would be required to be of greatest help to music therapists when using technology in the future, which might lead to more successful integration of technology into the practice of MT.

## Materials and Methods

### Recruitment

To recruit volunteers, an email including the relevant background, study motivation, and information regarding the online survey, was sent to MT organizations in Asia, Europe, and North America via their website contact form or to the organization’s secretariat to recruit potential participants.^1^ In addition, information was distributed on social media (i.e., music therapy Facebook groups, such as the World Federation of Music Therapy). Volunteers were allowed to share the survey by word of mouth (i.e., to their music therapist contacts), and the information and survey link were also shared with music therapists who were personal contacts of the study investigators. These forms of advertisement resulted in participants from several additional countries. Only certified music therapists who were currently practicing MT were invited to be a part of the study.

### Survey Procedure

At the beginning of the survey, an introduction to the study was displayed via the online form, describing the aims of the study and how the information gathered would be used. Next, participants were asked to provide their informed consent before participating. If the participant gave consent, they could then proceed on to the survey questions; if not, they did not proceed. No names or other personally identifiable information were collected during the consent taking process. Participants were also informed that this research study was approved by the National University of Singapore Institutional Review Board (NUS-IRB Reference Code: NUS-IRB-2020-146).

The survey was provided in English and was divided into three sections. Participants could not continue to the succeeding sections if previous sections were not completed. The first section of the survey consisted of questions relating to the background of the music therapist and their practice, e.g., their demographic information, as well as their expertise in the field of MT and the type of MT conducted. The second section focused on their use of technology within their practice of MT, including their experience using technology, and more general views on the use of technology in MT (such as the benefits and limitations of technology use). The final section dove into the impact of COVID-19 on their MT practice, namely, how their MT practice was altered during this period of time, and which online/virtual tools were utilized to enable the continuation of their practice (when this was possible at all). Looking toward the future and the possibility of embracing greater technology use, music therapists were also asked whether more training opportunities would be welcome, and generate increased adoption of technology in the future. At the end of the survey, participants were given the option of entering their email address to participate in the prize drawing of one of two US$50 amazon gift vouchers, as a token of appreciation for participating in the study. In total, there were 36 questions within the survey, across the three sections described above, and the survey took approximately 15–20 min to complete.

Participants completed the survey during the month of September in 2020, several months after the COVID-19 pandemic swept across the world, and after all of the countries contacted went through (or were still in middle of) some type of lockdown or social distancing period. Once all responses were made, we examined the aggregate quantitative data, as well as qualitative (short answer) responses, that were collected from the survey. The results are provided in section “Survey Results and Discussion.”

### Participants

A total number of 112 volunteers participated in this study, hailing from three different regions around the globe: North America (including Canada and the United States), Europe (including Austria, Belgium, Germany, Italy, Lithuania, Luxembourg, Netherlands, Spain, and Switzerland), and Asia/Oceania (including Australia, Bahrain, Hong Kong, Indonesia, Israel, Malaysia, New Zealand, Singapore, Taiwan, and Thailand). Of those who participated, 16.1% identified as male, 81.3% identified as female, and 2.7% identified as non-binary. In terms of age, 23.2% of the participants were between 20 and 29 years of age, 31.3% were between 30 and 39 years, 25.0% were between 40 and 49 years, 13.4% were between 50 and 59 years, and 7.1% were aged 60 or above.

## Survey Results and Discussion

Here we discuss the results of the three main sections of the survey as described in the section “Survey Procedure.” First, the participants’ professional background is presented, which covers the music therapist’s training, qualifications, type of MT conducted, and general characteristics about their patients. Second, we present music therapists’ responses about their use of technology in their MT practice. Third, we present responses about how the music therapists’ have had to change and adapt their practice during the COVID-19 era, and also how their use of technology has changed accordingly. We further discuss what the therapists suggest might be the most helpful features of technology to consider moving forward, including factors both inherent to and external to the technology (such as affordability), and how a more widespread use of technology may be adopted through educational workshops and training sessions.

### Background Questions

The demographic background of the music therapists who completed our survey is provided in the section “Participants.” This section provides details regarding the professional background and experience of the participants, and the types of patients the music therapists see in their practice.

The participants had an average of 10.8 years (SD = 9.4 years) of professional experience as a music therapist. In terms of their MT qualifications, 26 participants (23.2%) indicated holding a Diploma in Music Therapy from a private institution, 35 (31.3%) hold a Bachelor’s degree in Music Therapy, 54 (48.2%) hold a Master’s degree in Music Therapy, 4 (3.6%) have a Doctorate degree, and 13 (11.6%) indicated other degree qualifications. The majority of music therapists conduct both group and individual MT sessions (74 individuals, or 66.1%), while 25% conduct individual sessions only, and 8.9% conduct group sessions only. In terms of the method of MT conducted, the majority conduct both active and receptive MT (83 individuals, or 74.1%), while 23.2% conduct active MT only, and 2.7% conduct receptive MT only.

In terms of the background of the patients seen by our sample of music therapists, there was a relatively even distribution of patients from each age range, from 0 to 9 up to 60+ years of age. Details of the age ranges of patients seen can be found in Table 1(a). There was also a wide range of patient diagnoses seen by music therapists. Because the question about patient diagnoses (“What clinical needs/diagnoses (motor impairment, aphasia, etc.) do your patients/patients tend to have?”) was open ended, responses were categorized into patients from more than one diagnostic category. The most prevalent diagnostic category of patients was *neurodevelopmental disorders*, and the full distribution may be found in Table 1(b). The percentages in Table 1(b) provide the percentage of music therapists from a region who have treated that particular diagnostic category (for example, 13% of the music therapists from Europe treat patients with *trauma and stress related disorders*). Note that because many music therapists indicated treating more than one diagnostic category, the percentages across each row total more than 100% for each region.

**TABLE 1:** Overview of general patient characteristics.

(a) Breakdown of age range of patients seen by music therapists.	
**What is the age range of your patients?**					**Total number of responses across all music therapists**		

0–9					56 (50%)		
10–19					61 (54.5%)		
20–29					60 (53.6%)		
30–39					53 (47.3%)		
40–49					50 (44.6%)		
50–59					54 (48.2%)		
60+					67 (59.8%)		

**(b) Breakdown of patient diagnoses seen by music therapists (across all regions, and broken down by region), where TSD = Trauma and stress related disorders, NDD = Neurodevelopmental disorders, DCD = Disruptive and Conduct Disorders, PD = Psychiatric Disorders, NCD = Neurocognitive Disorders, MED = Medical condition, and LIFE = Lifespan care.**							

	**TSD**	**NDD**	**DCD**	**PD**	**NCD**	**MED**	**LIFE**

**All**	19	63	9	37	32	11	13
Europe	7 (13.0%)	22 (40.7%)	5 (9.3%)	30 (55.6%)	12 (22.2%)	6 (11.1%)	4 (7.4%)
North America	6 (19.4%)	18 (58.1%)	3 (9.7%)	4 (12.9%)	10 (32.3%)	3 (9.8%)	4 (12.9%)
Asia/Oceania	6 (22.2%)	23 (85.2%)	1 (3.7%)	3 (11.1%)	10 (37.0%)	2 (7.4%)	5 (18.5%)

*Note that music therapists often indicated treating more than one diagnosis; therefore, percentages across the row total to more than 100% for each region.*

### General Questions About the Use of Technology in One’s Practice of MT

In order to be able to compare the participants’ technology use prior to and during the pandemic, we first asked the music therapists general questions about their use of technology. These responses are reported and discussed below.

#### General Attitudes and Use of Technology

When asked “Do you currently use technology (broadly defined) within your practice of music therapy?”, 96 participants (85.7%) responded “Yes,” while 16 (14.3%) responded “No.” This is in line with the rapidly increasing trend of greater technology use, from Magee (2006), in which 30% of music therapists responded having used (electronic) music technology in their clinical work, to Hahna et al. (2012), reporting just six years later that 71% of those surveyed had used music technology in a clinical setting.

In a follow-up optional question, we asked “If you do not use technology, do you believe that the use of technology could help you conduct music therapy sessions in the future?” Here, 52 reported “Yes” (85.2% of those who responded), 5 (8.2%) responded “No,” and 4 (6.6%) reported “Other” or “It depends.” This displays a strong positive view toward technology, and an openness about embracing technology for MT in the future.

To gain a more precise view of the specific technologies that music therapists have used in the past, as well as those which would be most beneficial to use (that are not currently employed) in the future, we asked about a set of the specific technologies, ranging from specialized MT software to more general technologies such as recording technologies and wearable devices. These findings are reported in Table 2.

**TABLE 2:** Overview of technologies used, and those that would be beneficial in the future (total number of affirmative responses across participants is provided in both columns).

Type of Music Technology	What technologies have you previously used during music therapy sessions?	What types of technology would be most beneficial to your practice of music therapy that you do not currently use?
Specialized Music Therapy Software	3	59
Apps	68	22
Streaming Service	58	19
Electronic Hardware/Devices	56	15
Music Software, including Compositional and Mixing Tools	41	31
MIDI Instruments	19	28
Teleconferencing Tools	54	25
Recording Technologies	76	28
Virtual Instruments	28	43
Smart Devices (smartphones/tablets)	87	19
Wearable Devices	13	22
New digital musical interfaces	3	28
Brain Computer Interface (BCI) technology	1	21
Serious Games (Games not designed purely for entertainment, that focus on education, healthcare, etc.)	10	19
Other	10	2
None	5	11

As one can see, many out of this pool of music therapists have used smart devices, apps, recording technologies, as well as streaming services, electronic devices, and teleconferencing tools. Few have used specialized MT software, new digital musical interfaces, or brain computer interfaces (BCIs). In terms of the technology viewed most beneficial to use in the future, over half of the respondents (59 individuals, or 52.7%) reported that specialized MT software would be the most useful for their practice. This was followed by virtual instruments, music software, and MIDI instruments/recording tools/new digital musical interfaces. Twenty-one (19%) or more of the respondents were also interested in tele-conferencing tools, apps, wearable devices and BCI technology as well.

In addition to being asked which kinds of technologies would be useful in the future, music therapists were also asked, “To be worth using in your MT practice, what would technology need to offer?” (see Table 3). These open-ended responses were categorized according to topic(s) mentioned multiple times (i.e., by more than one music therapist); note that many music therapists mentioned several aspects of what technology would need to offer to be worthwhile. According to our respondents, the most important factors are “Ease of use,” “Cost Effectiveness and affordability,” and “Accessibility/availability of technology.” These were followed by “Hardware/software to support patient’s needs,” and the ability to “Assist with analyzing patient’s performance or diagnosis.” Of lesser concern, but still noteworthy, were points regarding “Effectiveness, security, and reliability,” and aspects concerning a high-quality online experience, such as stable wifi, minimizing the internet/online time-lag, and aspects that support the online music-making experience.

**TABLE 3:** What technology would need to offer to be worth adopting?

To be worth using in your MT practice, what would technology need to offer? (e.g., ease of use, ability to analyze patients’ performance in new ways, hardware/software to support patient’s needs, accessibility/availability of the technology, cost-effectiveness/affordability, etc.)	
Ease of use	58
Assist with analyzing patient’s performance or diagnosis	21
Hardware/software to support patient’s needs	26
Accessibility/availability of technology	48
Cost-effectiveness and affordability	56
Good online experience: consistent wifi, no issues with internet time lag, and support for online music-making	12
Wearability/portability	3
After-purchase support	3
Improve quality of experience or music delivery, is not invasive to the experience	9
Engaging	2
Fills a need/gap/adds something additional to the experience	9
Effectiveness, security and reliability	14
Other	8
Not applicable	1

#### The Use of Technology for Assessment

As opposed to using technology to help conduct MT more generally, music therapists have recently been increasingly interested in using technology for the diagnosis or repeated assessment of patients. Along these lines, we sought to explore whether our sample has used technology for assessment purposes. When asked “How does technology influence your assessment of patients?”, 50% of respondents did not provide a response or did not understand the question (note that this was an optional question, as not all music therapists use technology when assessing patients). Of the remaining 56 participants, 30 individuals (53.6% of those who responded) indicated that the use of technology improves some aspect of the quality of observation of patients (feasibility, usability, accuracy, widening the scope of assessment, less time-consuming, etc.), and 9 individuals (16.1% of those who responded) indicated that the use of technology increases engagement.

#### Limitations of Technology

Music therapists were asked, “What limitations have you encountered when using technology in your music therapy practice?” Here, 49.1% (*n* = 55 of 112) of music therapists mentioned *limitations relating to the use of technology when conducting online sessions*, 37.5% (*n* = 42 of 112) described *limitations when using technology during in-person sessions*, and 13.4% (*n* = 15 of 112) did not provide an answer. Below, we first describe the limitations of conducting online MT, and then we describe the limitations of technology during in-person sessions, as described by the respondents.

In total, 76.4% (*n* = 42 of the 55 respondents who described limitations of technology in online sessions) indicated that conducting online sessions “interfere with the ongoing therapeutic process.” Among these 42 therapists, many (*n* = 21 of 42; 50%) found that the lack of physical contact, and “not being in the same room,” affected their therapeutic listening attitude and their capacity to know the right timing for specific interventions. The lack of close physical proximity and non-verbal cues made it difficult to attune to and empathize with the patient. This difficulty is associated with a loss of richness and vitality in (inter)subjective experiences, and a decrease in interaction and engagement, as shown in patients who are more easily tired and distracted. Other therapists (*n* = 11 of 42; 26.2%) pointed to the latency time, the unpredictable internet connection, and poor sound quality as disturbing, particularly in making it difficult to play together and simultaneously with the patient. In addition, some of the therapists (*n* = 8 of 42; 19%) described how they experienced, when confronted with non-familiar technology, a lack of necessary skills and training. This restricted them in their capacity to guide and support the patient. Finally, some therapists (*n* = 3 of 42; 7.1%) mentioned limitations with regard to the restricted field of vision. A screen camera allows only one perspective, i.e., there are fewer degrees of freedom to observe each other, which reduces the potential to target multiple modalities in therapy. Moreover, the overall image or sense of the group is lacking; that is, there is no group in its totality, because only a few people are visible at once.

In addition, 54.5% (*n* = 30 of the 55 respondents who described limitations of technology in online sessions) indicated problems related to the “accessibility of technology,” and described that this complicated the organization of online sessions. Among these 30 therapists, most of them mentioned difficulties because of a poor or unreliable internet connection (*n* = 24 of 30; 80%) and unstable applications (*n* = 6 of 30; 20%). Others (*n* = 10 of 30; 33.3%) stated that they did not have sufficient access to (specialized) devices and software for their sessions. Respondents mentioned that this lack of access was due to financial issues, but also because devices and software were not adapted to the needs of patients with limited mobility. They described this as a limitation, especially during the COVID period, where online MT was the only possibility to stay in touch with patients. It is also worth noting that one respondent (*n* = 1 of the 55 respondents who described limitations of technology in online sessions) experienced no limitations during online sessions, and mentioned explicitly the importance of online sessions to get in touch with isolated patients in times of crisis.

As mentioned above, 37.5% (*n* = 42 of 112) of the music therapists described limitations when using technology during in-person sessions. Of these respondents, 52.4% (22 of the 42 respondents who described limitations of technology during in-person sessions) mentioned that technology “interferes with the ongoing therapeutic process” during in-person sessions. Some of these 22 therapists (*n* = 5 of 22; 22.7%) describe how technology hinders human connection and affective attunement, and how it is sometimes used as a digital wall to avoid interaction and fears. Others (*n* = 8 of 22; 36.4%) mention that patients are more distracted, decreasing the development of interpersonal functioning and regulation when using technology in sessions. Finally, respondents (*n* = 9 of 22; 40.9%) describe limitations due to a lack of necessary skills and training, which restricts them from being able to work flexibly and attune themselves to the patients’ needs or solve problems when necessary.

Many therapists (50%, or *n* = 21 of the 42 respondents who described limitations of technology during in-person sessions) indicated problems related to the “accessibility of technology,” such as poor or unreliable internet connection (*n* = 5 of 21; 23.8%), unstable applications (*n* = 9 of 21; 42.9%), and no access to specialized devices and software for their sessions due to financial issues (*n* = 6 of 21; 28.6%), but also because devices and software were not adapted to the specific needs of patients (*n* = 7 of 21; 33.3%). Finally, 14.3% (*n* = 6 of the 42 respondents who described limitations of technology during in-person sessions) mentioned “no limitation” when using technology in their MT practice.

#### Views Toward Technology by Geographical Region

To our knowledge, no survey of the use of technology in MT has examined regional differences, especially in non-Western countries, beyond comparing the United States to “non-US countries” including Australia, Canada, and the United Kingdom (see Hahna et al., 2012). Because we were able to collect responses from music therapists from 21 countries in three regions around the world, and because views toward technology use may vary given different MT traditions and broader cultural differences, we sought to investigate whether any regional differences exist in these attitudes and trends. Therefore, we examined the use of technology across three regions—Europe, North America, and Asia/Oceania—as outlined above. In this section, we provide an overview of the attitudes toward and use of technology in MT from these three regions.

Table 4 presents the results of the five questions in this section of the survey (regarding attitudes toward and actual use of technology) that were presented in Yes/No/Other format, broken down by geographical region. Note that the percentages reflect the responses by row, i.e., the percentage of Yes/No/Other by region. Generally, there were very positive views on the use of technology for improving patient outcomes, although this varied somewhat by region (See Table 4).

**TABLE 4:** General use of technology within music therapy.

Question	Yes	No	Other
**Do you currently use technology (broadly defined) within your music therapy practice?**			
All	96 (85.7%)	16 (14.3%)	
Europe	40 (74.1%)	14 (25.9%)	
North America	31 (100.0%)	0 (0.0%)	
Asia/Oceania	25 (92.6%)	2 (7.4%)	

**If you do not use technology, do you believe that the use of technology could help you conduct music therapy sessions in the future?**			
All	52 (85.2%)	5 (8.2%)	4 (6.6%)
Europe	27 (77.2%)	4 (11.4%)	4 (11.4%)
North America	18 (100.0%)	0 (0.0%)	
Asia/Oceania	17 (94.4%)	1 (5.6%)	

**Do you believe that the use of technology could help your patient more effectively reach his/her goals and outcomes across music therapy sessions?**			
All	84 (75%)	28 (25%)	
Europe	37 (68.5%)	17 (32.5%)	
North America	29 (93.5%)	2 (6.5%)	
Asia/Oceania	18 (66.7%)	9 (33.3%)	

**In regard to any training required in order to learn about and adopt new technologies, would you be willing to invest your time to this end?**			
All	101 (90.2%)	11 (9.8%)	
Europe	46 (85.2%)	8 (14.8%)	
North America	30 (96.8%)	1 (3.2%)	
Asia/Oceania	25 (92.6%)	2 (7.4%)	

**Do you believe that there would be greater use of technology within the practice of music therapy if music conservatories and music therapy schools offered more technology-specific training programs?**			
All	102 (91.1%)	10 (8.9%)	
Europe	46 (85.2%)	8 (14.8%)	
North America	31 (100.0%)	0 (0.0%)	
Asia/Oceania	25 (92.6%)	2 (7.4%)	

There are several noteworthy findings here. First, there were differences in technology use by region: All of the music therapists from North America indicated that they currently use technology (broadly defined) in their MT practice, and 92.6% of those in Asia/Oceania use technology. In contrast, 74.1% of music therapists in Europe reported using technology. A chi-square test confirmed that this difference between regions in technology use is statistically significant [χ^2^ (2, 112) = 15.8, *p* < 0.001]. For the following (optional) question about whether technology that is not currently used could be helpful to conduct MT sessions in the future, 100% of those who responded from North America indicated that it would be helpful, 94.4% responded positively from Asia/Oceania, and 77.2% of those from Europe responded affirmatively. There was no significant difference in this response across regions [χ^2^ (4, 61) = 6.97, *p* = *n.s.*]. These are very positive responses across all regions, although Europe’s enthusiasm for technology is slightly more tempered than responses from North America and Asia/Oceania.

A more obvious division in views is observed with the third question: “Do you believe that the use of technology could help your patient more effectively reach his/her goals and outcomes across music therapy sessions?” While there are once again very positive views from North America, only around two-thirds of respondents from the other regions responded “Yes,” and this difference was statistically significant [χ^2^ (2, 112) = 9.49, *p* < 0.01].

When asked about technology training (the fourth and fifth questions in Table 4), there were again very pro-technology views from all regions, with North America being the most embracing of technology, and Europe displaying the most cautious views. Specifically, when asked about whether they would be willing to invest their time in training and learning about new technologies, participants from all regions indicated overall that they would be willing to invest their time to this end, with 96.8% positive responses from North America, 92.6% positive responses from Asia/Oceania, and 85.2% positive responses from Europe. There was no significant difference between countries [χ^2^ (2, 112) = 3.54, *p* = *n.s.*]. When asked if they “believe that there would be greater use of technology within the practice of music therapy if music conservatories and music therapy schools offered more technology-specific training programs?”, there was unanimous (100%) agreement among North American music therapists, 92.6% concurring from Asia/Oceania, and 85.2% agreeing from Europe. This difference between regions was marginally significant [χ^2^ (2, 112) = 9.49, *p* < 0.05]. These views indicate that there is a fairly global pro-technology trend, with music therapists from all regions generally agreeing that technology would be useful for their practice, and that they would be willing to invest the effort to undergo some sort of training in order to be better prepared to adopt new technologies in the future (although European music therapists were more reluctant than others to invest their time in technology-related training).

When further asked about training opportunities (“Have you received training opportunities for incorporating technology into your practice, or has your experience using technology been self-taught (including consulting online references/sources?”; see Table 5), the majority of respondents indicated that they had not received training, although regional differences were apparent. Specifically, while around one quarter (25.8% of those from North America, and 25.9% of those from Asia/Oceania) reported having received training, only 5.5% from Europe have received training. This difference was statistically significant [χ^2^ (4, 112) = 12.62, *p* < 0.05]. Around two-thirds of respondents from all regions indicated being self-taught with respect to their use of technology, and the highest percentage of those with no technology-related training (27.8%) were from Europe. This finding closely matches previous findings, in which 61% of music therapists across the United States, United Kingdom, Australia, and Canada reported being self-taught with respect to music technology (Hahna et al., 2012).

**TABLE 5:** Training opportunities by region.

	Received training	Self-taught	No technology- related training
**Have you received training opportunities for incorporating technology into your practice, or has your experience using technology been self-taught (including consulting online references/sources)?**			
All	18 (16.1%)	73 (65.2%)	21 (18.7%)
Europe	3 (5.5%)	36 (66.7%)	15 (27.8%)
North America	8 (25.8%)	20 (64.5%)	3 (9.7%)
Asia/Oceania	7 (25.9%)	17 (63.0%)	3 (11.1%)

### Changes to MT Practice and to Use of Technology During the COVID-19 Era

To gain insight into how the music therapists’ practice and use of technology changed specifically due to COVID-19, we asked about the general changes needed due to the pandemic, as well as technology-specific questions, in the final section of the survey. Here, open-ended questions were included to gain a deeper understanding of music therapists’ experiences than is possible in the more superficial yes/no questions. The open-ended questions were analyzed using the “six-phase approach” of thematic analysis (i.e., familiarization, generating codes, constructing themes, revising, defining themes, and writing up) (Braun et al., 2019). An inductive coding approach was chosen where the active and reflexive process of the researcher was given priority during the qualitative analysis; as such, the results of this analysis will inevitably bear the mark of the researcher.

We first discuss the most broad and open-ended question, which was, “How has your music therapy practice changed due to Covid-19?” One hundred nine of the therapists (*n* = 109 of 112; 97.3%) indicated that their MT practice changed due to COVID-19. Two of these 109 (*n* = 2 of 109, or 1.8%) described changes related to their job function, i.e., being required to conduct tasks other than MT, or being moved to another unit. Thirty-five (*n* = 35 of 109, or 32.1%) mentioned that MT was not provided anymore, mainly due to (temporary) institutional decisions made in order to follow strict safety measures during the lockdown. Respondents described how these decisions affected both therapists and patients. Several therapists lost their job or were required to work only at one workplace. Many patients were no longer able to receive MT, which was described by two respondents as having drastic consequences, especially in end-of-life care.

Therapists who were able to continue practicing MT described how their MT practice had changed due to COVID-19. In this sample, 84 (*n* = 84 of 109, or 77%) of the respondents described changes to the MT setting. Many individuals reported that face-to-face sessions were no longer provided, leading to a need to adapt and search for alternative ways to maintain a connection with the patient via phone calls, streaming sessions, online MT sessions or telerehabilitation sessions. But even in cases where face-to-face sessions were maintained, or were rebooted after a strict lockdown, respondents mentioned many changes to the setting, related to specific safety rules (such as wearing a face mask, disinfection, and distance rules), and this was also associated with changes to the organization of the session, i.e., smaller groups, more individual MT, and shorter sessions. Some therapists described how they were no longer allowed to use musical instruments, and were especially forbidden from singing. Seventeen respondents (*n* = 17 of 109, or 15.6%) described how this change of setting influenced their MT method and interventions. Therapists elaborated here upon three themes: (i) the need to change to receptive methods in MT; (ii) the changing role of the therapeutic relationship; and (iii) the changing role of embodied expression and verbal language in interventions. Nine respondents (*n* = 9 of 109, or 8.3%) discussed how this change of setting had an impact on the MT culture in their sessions. Respondents elaborated here on three themes as well: (i) changes in the group culture in group sessions; (ii) changes in the subjective experiences of patients (e.g., a decrease in felt safety and security; increased neediness, dependency and depressive states); and (iii) changes in the quality of the therapeutic relationship. Finally, six of the respondents (*n* = 6 of 109, or 5.5%) described how the crisis also offers opportunities to be creative and flexible to explore new ways of conducting MT. This was related to exploring and learning how technology can be used in MT practice.

We also asked specific questions regarding how the music therapists’ practice has changed, and how their use of technology has changed, during the pandemic: in total, we asked nine questions in Yes/No/Other format, the results of which are reported in Table 6. Note again that the percentages reported reflect responses by row, i.e., the percentage of Yes/No/Other responses according to each region. The results of these nine questions, as well as an additional question reported in Table 7 on the effectiveness of online MT, are discussed in the following two subsections: the first discusses the ways that conducting MT has changed as a result of the pandemic, from reductions in patients seen to the consequences of conducting online (teletherapy) sessions, and the second section focuses specifically on technology use during COVID-19.

**TABLE 6:** Changes to MT practice and Use of Technology during the COVID-19 Era.

Question	Yes	No	Other
**During this period of COVID-19, do you believe that there is generally a greater need for music therapy?**			
All	97 (86.6%)	15 (13.4%)	
Europe	43 (79.6%)	11 (20.4%)	
North America	31 (100.0%)	0 (0.0%)	
Asia/Oceania	23 (85.2%)	4 (14.8%)	

**Have you seen fewer patients during the past several months due to COVID-19 than in the previous months/years?**			
All	90 (80.4%)	22 (19.6%)	
Europe	42 (77.8%)	12 (22.2%)	
North America	28 (90.3%)	3 (9.7%)	
Asia/Oceania	20 (74.1%)	7 (25.9%)	

**Have you needed to conduct teletherapy (virtual) sessions?**			
All	75 (67%)	37 (33%)	
Europe	26 (48.1%)	28 (51.9%)	
North America	29 (93.5%)	2 (6.5%)	
Asia/Oceania	20 (74.1%)	7 (25.9%)	

**If you have conducted online (teletherapy) sessions, did your patients ever use a musical instrument(s) they had available at home during the sessions?**			
All	48 (62.3%)	29 (37.7%)	
Europe	12 (42.9%)	16 (57.1%)	
North America	24 (82.8%)	5 (17.2%)	
Asia/Oceania	12 (60.0%)	8 (40.0%)	

**Furthermore, through your experience of administering music therapy online, has your practice been hindered by the remote setting between patient and therapist?**			
All	56 (76.7%)	17 (23.3%)	
Europe	19 (79.2%)	5 (20.8%)	
North America	22 (75.9%)	7 (24.1%)	
Asia/Oceania	15 (75.0%)	5 (25.0%)	

**Due to Covid-19, have you needed to embrace new techniques/technologies in order to conduct music therapy sessions?**			
All	78 (69.6%)	34 (30.4%)	
Europe	26 (48.1%)	28 (51.9%)	
North America	31 (100.0%)	0 (0.0%)	
Asia/Oceania	21 (77.8%)	6 (22.2%)	

**Has embracing more technology influenced your opinion of its use in music therapy?**			
All	57 (50.9%)	25 (22.3%)	30 (26.8%)
Europe	19 (35.2%)	15 (27.8%)	20 (37.0%)
North America	25 (80.6%)	5 (16.1%)	1 (3.2%)
Asia/Oceania	13 (48.1%)	5 (18.5%)	9 (33.3%)

**If you have conducted online (teletherapy) sessions, did you ever use virtual instruments or specialized software (not including conferencing software such as Skype) due to the virtual format?**			
All	18 (24.0%)	57 (76.0%)	
Europe	6 (23.1%)	20 (76.9%)	
North America	7 (24.1%)	22 (75.9%)	
Asia/Oceania	5 (25.0%)	15 (75.0%)	

**Will you continue using new technology or technology-related methods in the future that you embraced during the Covid-19 pandemic?**			
All	71 (63.4%)	23 (20.5%)	18 (16.1%)
Europe	26 (48.2%)	19 (35.2%)	9 (16.7%)
North America	25 (80.6%)	2 (6.5%)	4 (12.9%)
Asia/Oceania	23 (85.2%)	2 (7.4%)	2 (7.4%)

**TABLE 7:** Effectiveness of online therapy compared to real-life therapy.

	More	Less	As
	effective	effective	effective
**Based on your personal experiences, has conducting therapy sessions using online tools been more, less, or just as effective as real-life sessions?**			
All	1 (1.2%)	53 (63.9%)	29 (34.9%)
Europe	0 (0.0%)	26 (60.0%)	5 (40.0%)
North America	1 (3.4%)	13 (44.8%)	15 (51.8%)
Asia/Oceania	0 (0.0%)	14 (60.9%)	9 (39.1%)

#### Regional Differences in Changes to Music Therapy Practice During COVID-19

To gain a sense of the potentially increased need for MT during the pandemic, as well as whether (and how) music therapists were able to see patients and do their best to meet that need, we asked whether they believe there is a greater need for MT during the COVID-19 period (see Table 6). The response was largely affirmative, with 100% of those from North America agreeing that there has been an increased need for MT during the pandemic, followed by 85.2% of those from Asia/Oceania, and 79.6% of those from Europe. A chi-square test confirms that these results by region are statistically significant [χ^2^ (2, 112) = 10.96, *p* < 0.01]. When asked whether they have seen fewer patients during the past several months due to COVID-19, respondents reported that in fact, they had been forced to see fewer patients: 90.3% of those from North America confirmed seeing fewer patients, followed by 77.8% of those from Europe, and 74.1% of those from Asia/Oceania. The responses by region were not statistically significant [χ^2^ (2, 112) = 3.15, p = *n.s.*]. This finding tells us that while there was greater need around the world for MT, due to all of the hardships and stressors stemming from the pandemic, music therapists were unfortunately not able to see as many patients during this time, most probably due to social distancing measures and temporary clinic closures.

We also asked, in open-ended format, for music therapists to explain their response to the question of whether they believe there is greater need for MT. Overall, 68 respondents (*n* = 68 of the 97 therapists who indicated that they do believe there is indeed a greater need for MT during the COVID-19 period; or 70.1%) discussed how MT is needed to provide social-emotional support and connection in times of social isolation. This is illustrated in the following quote from one respondent, writing about MT: “Music is always about making a connection, whether it is interpersonal or within oneself, and connection is precisely what we tend to lose during this time of isolation and quarantine.” The second most quoted reason (to explain the greater need for MT) was the need to target increased levels of stress and anxiety. Finally, respondents explain how the impact of the COVID-19 situation has led to an increased awareness both in stakeholders and policy makers of the importance of non-verbal and embodied qualities targeted in MT, and the role of these non-verbal qualities for well-being and quality of life.

We then asked several questions about whether the therapist has had to conduct online (teletherapy) sessions with their patients. Here, there was a large difference based on region: most of those from North America have needed to conduct online sessions (93.5%), as opposed to three-quarters (74.1%) of those from Asia/Oceania, and fewer than half (48.1%) of therapists from Europe. These differences are statistically significant [χ^2^ (2, 112) = 21.59, *p* < 0.001]. The following two questions aimed to gain more clarity about the nature and limitations of online therapy in those who did conduct virtual sessions.

When asked whether their patients ever used musical instruments they had available at home, 82.8% of respondents from North America, 60.0% from Asia/Oceania, and 42.9% from Europe responded affirmatively. This difference by region was statistically significant [χ^2^ (2, 112) = 10.18, *p* < 0.01]. These percentages suggest that while the majority of patients seen in North America had a physical instrument available to support their online MT session, many fewer were in this position in Asia/Oceania, and potentially half of those had an instrument available (compared to North America) in Europe. We speak about the use of online tools and instruments in the following section.

Music therapists were also queried as to whether the remote setting between patient and therapist during online sessions has hindered their MT practice. Here, based on those who responded to this optional question, between 75 and 79% of music therapists from all regions reported “Yes.” A chi-square test confirmed no regional differences [χ^2^ (2, 73) = 0.13, *p* = *n.s.*]. Therefore, we can infer that roughly three-quarters of music therapists in these regions around the world have found online sessions to be sub-optimal, as compared to in-person sessions. In an optional question, we also asked music therapists if they believe that conducting MT using online tools has been more, less, or equally effective compared to real-life sessions (see Table 7). The majority of respondents reported that online MT has been less effective, although just over half (51.8%, or *n* = 15 out of 29) of those from North America found online MT to be just as effective as in-person sessions. In contrast, only around 40% of those from Europe and Asia found online MT to be as effective as real-life sessions. These regional differences were statistically significant, [χ^2^ (4, 83) = 11.8, *p* < 0.05]. Together, these results inform us that the respondents to this study would largely prefer their practice to be face-to-face when possible, and suggest that tele-music-therapy (TMT) may not be widely embraced in the future due to its drawbacks and limitations. This finding is in line with several limitations discussed in the literature, namely that remote sessions reduce the opportunity for affective, embodied, interpersonal experiences between the patient and therapist, and that they raise concerns regarding patient well-being, such as increased anxiety due to limited privacy of the sessions (e.g., Desmet, 2020).

From these findings, one can see that online sessions are very often regarded as less effective than in-person sessions, and seen as just as effective in the best case scenario. For further discussion of the effectiveness of online MT sessions, please refer to section “Limitations of Technology.”

#### Regional Differences in Technology Use During COVID-19

Here we discuss the results of questions 6–9 in Table 6, which focus on the technology that music therapists have adopted during the pandemic. First, we found that the majority of music therapists have needed to embrace new technologies during COVID-19, as expected, however there were large differences based on region, which were statistically significant [χ^2^ (2, 112) = 34.11, *p* < 0.001], with 100% of respondents from North America reporting that they have had to embrace new technologies/techniques in order to conduct MT sessions, in comparison to 77.8% of those from Asia/Oceania, and only 48.1% from Europe. There were also striking differences between North America and the other regions when asked whether embracing new technologies has influenced the therapist’s opinion of the use of technology in MT. Here, 80.6% of those from North America concurred with this statement, while only 48.1% of those from Asia/Oceania, and 35.2% of those from Europe, agreed with the statement. These differences are statistically significant [χ^2^ (2, 112) = 21.66, *p* < 0.001].

The music therapists were asked whether, during online sessions, they ever used virtual instruments or specialized software (not including conferencing software such as Skype) due to the virtual format. About one quarter of the therapists from each region responded that they had used virtual instruments or specialized software, ranging from 23% in Europe to 25% in Asia/Oceania, and this result was not different across regions [χ^2^ (2, 75) = 0.02, p = *n.s.*]. This suggests that while most music therapists did not use virtual instruments or speciality software, there were a considerable number of therapists around the world who have tried using special tools to support the online MT format. Finally, the majority of music therapists in North America (80.6%) and Asia/Oceania (85.2%), but not Europe (48.2%), said that they will continue to use technologies that they adopted during the pandemic. This difference is statistically significant [χ^2^ (4, 112) = 18.22, *p* < 0.01]. This is interesting to consider given the general reluctance toward technology exhibited more overtly by European music therapists compared to the others (described in section “Limitations of Technology”), and the finding above that less than half of the European therapists have adopted new technologies in order to conduct MT sessions during COVID-19.

## Conclusion

### Takeaway Messages and Concluding Remarks

The results from this survey offer a rich set of findings regarding the practice of MT during COVID-19, describing how music therapists have had to alter their practice, and providing insight into when and why new technologies have been adopted during this time. Because therapists from over 20 countries around the world responded to this survey, it was possible to explore differences in views and approaches to MT between Asia, America, and Europe. That is, not only was the general impact of the pandemic on the practice of MT (and incorporation of technology) of interest, but also the differential effects based on region.

First, in terms of the most general findings regarding the use of technology in the practice of MT, the majority of music therapists in this sample indicated that they have used some form of technology in their practice. This is in line with an increasing trend in technology use in MT, given the findings from Magee (2006) and Hahna et al. (2012). That said, the increasing use of technology does not appear to be ubiquitous, but depends somewhat on the region in which one practices MT.

There were significant differences across regions in terms of whether the music therapists use technology in their practice: almost all respondents from North America and Asia/Oceania have used technology, while around three-quarters of those from Europe have used technology. A similar pattern—of more positive views from North America and Asia/Oceania compared with Europe—was demonstrated for the questions about whether technology can help patients more effectively reach his/her goals and outcomes.

It is interesting to note that the highest percentage of those with no technology-related training were from Europe, and those from Europe were also the most hesitant to claim that the use of technology would be beneficial for future MT sessions, or agree that technology may enable patients to more effectively reach their clinical goals. It may be that fewer training opportunities and less overall familiarity with technology have contributed to greater skepticism in this region regarding the efficacy of technology and willingness to embrace technology for future use, in comparison to North America and Asia/Oceania, where both training and positive views toward technology were more prevalent. It is also possible that differences in the cultural context and theoretical orientation across regions (along with the types of interventions that therapists are inclined to integrate into their practice based on their orientation) contributed to the differences seen across regions in attitudes toward and use of technology. Further, it is possible that many of the more skeptical music therapists are in fact interested in the use of technology, but the limitations of technology that they have encountered foster their reluctance, as respondents described a significant need for more human-, therapy- and relationship-driven technology, as opposed to the technology-driven methods that are more prevalent. That is, while many therapists are open to the notion of including technology and digitalization in their practice, especially during times of crisis and isolation, the majority of music therapists across all regions are concerned with losing basic, affective and embodied modalities in human interaction that they view as so vital to therapy. These humanistic, embodied aspects are often described by respondents as being at the center of the therapeutic relationship. One may therefore speculate that it is not only the differences in training opportunities and prior use (as well as MT methods, traditions, and approaches) that are responsible for different viewpoints regarding technology use, but also cultural differences, such as the importance of interpersonal interaction, and reluctance to conduct virtual therapy sessions.

The findings above discuss general trends and views in technology; next we review the findings related to COVID-19 in particular. In relation to changes to the practice of MT and adoption of technology due to the pandemic, nearly all (97.3%) of the music therapists in this study indicated that the pandemic has impacted their MT practice. Nearly one third of respondents indicated that their practice of MT was temporarily halted, and several therapists indicated that they had unfortunately lost their job. For those who were able to continue practicing MT, the majority discussed alternative approaches (such as phone calls, streaming sessions, and online sessions) they needed to adopt in order to maintain a connection with their patient. In addition, the vast majority of music therapists believe that there is currently a greater need for MT (especially regarding stress and mental health issues), but most therapists have seen fewer patients in the wake of COVID-19, due mainly to lockdown measures, social distancing, funding, and department-level changes during the pandemic.

Some countries and regions were harder hit, and faced stricter lockdown measures, than others. For example, the majority of music therapists in North America and Asia/Oceania have needed to conduct virtual MT sessions due to COVID-19, while less than half of those from Europe had to do so. Interestingly, the region with the most widespread reported use of technology in general (all of the music therapists from North America) also had the most positive responses (over half) regarding online sessions being just as effective as in-person sessions. This result (of positive views of online MT from North American music therapists) is generally in line with recent findings from the American Music Therapy Association: in their survey, the majority of respondents reported that their clients have had a positive response to remote MT sessions (Fay et al., 2020). That said, the majority of music therapists in our sample (between 75 and 79% across regions) who have had to conduct online sessions believed their sessions were hindered by the remote setting. It was also striking to find that those with the greatest amount of training and greatest adoption of technology (as well as the most prevalent use of teletherapy) were also the most optimistic about online sessions and about technology in general (see Tables 4, 6, 7). The implication is that technology-specific training may lead to greater use of technology, which may in turn lead to more optimistic opinions of technology use in MT, a point that we further explore in the next section.

We also investigated the ways in which the pandemic has altered the practice (setting, methods/approach, outcomes, and overall “feel”) of MT. The majority of music therapists reported that COVID-19 resulted in changes to the *music therapy setting*, such as needing to find new ways to stay in touch with their patients (online sessions, phone calls, etc.), organizational changes (such as more individual sessions), and restrictions on sharing instruments or singing when in-person sessions were possible. This is in line with a recent study from the United States showing that music therapists were remarkably resilient to adapt and shift to alternative delivery methods such as telehealth (Gaddy et al., 2020). While many music therapists in the study by Gaddy et al. (2020) were grateful to be able to offer virtual services to maintain contact with their patients, therapists also described the negative impact of the disruption of in-person sessions on their mental health and professional identity. This relates to a recent paper in which team members in a Belgian ward, offering group therapy for people with personality disorders and dysfunctions, describe how drastic transformations took place in the ward due to preventive COVID-19 measures (Van Duppen et al., 2021). They reflect:

In only a few days’ time, the ward had changed completely. What was once a therapeutic ward with long-standing routines, rhythms and continuity, had transformed into a hastily abandoned collection of empty hallways, unused chairs and soulless rooms. Patients, as fragments of their group and community, wandered about the ward, as confused testimonies of what was once a self-evident normality. Others were at home, no longer part of their group, increasingly forgotten by the team, pushed aside by the intrusive tasks of the next day. … We had set up a base camp: à la guerre comme à la guerre! (p. 21, translated from Van Duppen et al., 2021).

Some therapists in our sample also discussed needing to *change their MT method or intervention*, how the therapeutic relationship had changed, and how the roles of embodied expression and verbal language were impacted during the pandemic. They described how the musical affective-embodied attunement between therapist and patient, which forms the base for the relational context in which a new psychological balance may be reached and inner experiences may take shape, was disturbed. This disruption makes it difficult to truly be in the present moment with the patient (Stern, 2004). The therapists’ reflections on how their methods and embodied relationship with patients changed relates to the following impression of a music therapist (Van Duppen et al., 2021), which describes the impact of the COVID-19 measures during face-to-face sessions:

All musical instruments hang on the wall, decontaminated, no longer allowed to be played, leaving the chairs naked in the room. The murmur of patients echoes hollowly against the hardness of the static walls. The anticipating silence of a potential interplay, where music emerges, has degenerated into a desolate sound.

The time of the here and now, in which bodies seek to tune in, through which I let myself be guided musically along tension and relaxation, frustration and satisfaction, has been stripped of all pulsation. Without this pulsating process of *Gestaltung*, every movement, every act, seems to be a tenuous orchestration in a tightly conducted time. I feel awkward. I am voiceless, in a frantic attempt to resonate without senses. (p. 24, translated from Van Duppen et al., 2021).

In cases where patients were no longer allowed to come to the clinic or ward, this disruption to embodied attunement was especially apparent. Some therapists in our sample noted how patients were offered only a minimal degree of virtual or phone contact, and emphasized the limited access to affective embodiment in human interaction during online sessions.

Although many music therapists embraced new technologies out of necessity during COVID-19, there were large differences in many cases based on the region, with all respondents from North America indicating that they had recently adopted new technology to conduct MT, in comparison with a little over three-quarters of those from Asia/Oceania, and fewer than half of those from Europe. Around one quarter of respondents from each region indicated that they had used virtual instruments or specialized software when conducting online sessions during the pandemic. Compared to Europe, many more music therapists from North America and Asia/Oceania said that they will continue to use technologies that they adopted during the pandemic. With this view toward the future in mind, the final section of this paper focuses on the implications our findings have for how to move forward, and what might be able to benefit the practice of MT in the future.

### Implications for Music Therapy During Times of Crisis, Insights Gained About How to Move Forward, and Considerations for the Future

Here we draw conclusions from the findings of this study that are pertinent for the future of MT, and the incorporation of technology into the practice of MT in the future. Before discussing the findings and implications, it is pertinent to note here that the theoretical framework and applied methods of the music therapist will influence the needs, experience, and roles of technologies adopted for MT. In addition, sociocultural factors and the cultural context in which the therapy is administered will further influence the use of technology in MT. The role of the musical exchanges in the therapeutic relationship, and aspects such as the subjective experiences in the MT process, need to be considered when deciding when and whether to adopt particular technologies. With these, as well as patient needs and considerations in mind, therapists can assess what technology may be used both to promote the patient’s wellbeing, and to avoid a decline in the quality of human relationship involved in the MT process.

It is also prudent here to note the limitations of this study. First, given the number of countries involved, we had a relatively small sample size, due in part to the brief data collection window, and the fact that the survey was only administered in English. Note, however, that our sample is comparable to other studies of technology adoption in MT (e.g., Magee, 2006), and many of the differences between regions had relatively large effect sizes, which allow us to be more (cautiously) confident when making generalizations regarding regional trends. Another limitation is that during the data collection period (September 2020), many music therapists were still in the process of changing and adapting based on the repercussions of COVID-19 and social distancing measures. We hope that this work can serve as a useful snapshot of the technology use and changes that occurred due to the pandemic. Further, our study included several open-ended questions, and, as is the case with thematic analysis, distilling themes apparent in all of the free responses will reflect the researcher’s sensibilities to some degree (Braun et al., 2019). Finally, a thorough investigation of the values and beliefs of the therapists was outside of the scope of this work; however it would be interesting to explore this aspect (especially in relation to technology use and views toward remote therapy sessions) in the future.

In terms of using technology to support the practice of MT in the future, a large percentage (over 85% of those who responded) believe adopting technology would be useful for conducting MT in the future, and the majority of music therapists in North America and Asia/Oceania, but not Europe, said they will continue to use technologies they embraced during the pandemic. For the specifics of which technologies precisely, the survey provided not only a current breakdown of the software and hardware technologies used by music therapists around the world today, but also examined which technologies are viewed as most beneficial to adopt in the future. There appears to be a clear desire for more specialized MT software; indeed, music therapists often use software and devices that were created for different or more general purposes (e.g., recording technology, wearable devices, and BCI systems), and there is a lack of bespoke MT software available today. In the future, music therapists and technologists should collaborate to develop effective tools and techniques that maximize the patient’s wellbeing, and are minimally disruptive to the MT process and therapeutic relationship (Agres et al., 2021). Finally, to be worth adopting, music therapists indicated that they believe technology should be easy to use, affordable, and accessible for their patients. In addition, the technology should be able to support their patients’ needs, and ideally be able to help analyze the patients’ performance or assess their diagnosis/progress.

The limitations reported by music therapists also gave insight into the drawbacks of technology (both in online and in-person settings), and what would be helpful to improve in the future. In terms of the drawbacks of conducting online sessions, many music therapists reported difficulties from the lack of physical contact, such as a lack of non-verbal cues, lack of rich/subjective experience, and a decrease in interaction and engagement (as described above in section “Takeaway Messages and Concluding Remarks”). The open, therapeutic listening attitude, characterized by a receptive welcoming and affectively resonant listening, was strained. Consequently, the role and quality of the therapeutic relationship changed, transference relations crumbled, and therapists felt inadequate. Online therapeutic sessions were turned into mere moments of contact meant to offer continuity for the duration of the interruption (COVID-19 period), rather than a holding environment that could help to recover from the gaping hole of overwhelming rupture and loss.

Many other therapists mentioned basic issues with the communication medium itself, i.e., an unreliable internet connection. In addition, many therapists working remotely did not have sufficient access to specialized devices and software to help conduct their sessions, which made online MT sessions particularly challenging. This was primarily the result of both funding restrictions and the fact that software and devices were not adapted to the needs of patients. It is undeniable that teleconferencing platforms such as Zoom and Skype made online sessions possible in many scenarios where they would otherwise be impossible, but for remote MT sessions, there is a clear need to develop technology that is better able to support the embodied and affective experiences and benefits of in-person MT. For both online and in-person sessions, some music therapists mentioned that technology interferes with the ongoing therapeutic process, e.g., technology hinders human connection and affective attunement. These concerns need to be considered in future directions and technological developments. In order to design a synergetic practice, more research is needed to develop and integrate technology that is fundamentally human- and relationship-driven (Agres et al., 2021).

In addition to the need for more humanistic, bespoke technologies, another crucial factor for the future is specialized training. Indeed, a recent study indicated that about two-thirds of music therapists from a large sample around the world would like to have more formal training opportunities as part of their MT credentialing/certification program (Kern and Tague, 2017). The music therapists in our study mentioned that a lack of training has limited their knowledge and ability to use technology with patients during in-person sessions. Most music therapists (although slightly fewer from Europe) agreed that if MT schools and music conservatories offered more technology-specific training programs, that there would be greater adoption of technology within MT. To date, technology-specific training is scarce in the curricula of MT training courses. To move along in a meaningful way, it is advisable to train educators in MT first, increasing their knowledge about the current state-of-the-art. In addition, training for supervisors associated with a professional MT organization would be helpful, as they guide many therapists in their clinical work. While most music therapists in our sample have not received technology-specific training, therapists from all regions indicated that they would be willing to invest their time in training and learning about new technologies. Therefore, while music therapists around the world believe they have not had sufficient opportunities for technology-specific training, there is a clear desire for more training. MT organizations may help meet these needs of their members by organizing dedicated workshops and training.

Further, an interesting link between training and views toward/adoption of technology emerged in our results: those in our sample who received the fewest training opportunities (i.e., those in Europe) were also the least likely to use technology, and had the most reserved opinions about whether technology might prove helpful when conducting MT sessions in the future. The other regions, with higher percentages of those who had received training or were self-taught, held more optimistic views of technology in MT, and have adopted technology at higher rates. Therefore, additional training may lead not only to smoother technology use during in-person and online MT sessions in the future, but would likely enable greater adoption of a wider range of technologies moving forward.

Alongside more formal training opportunities, the formation of peer-mentor groups (involving international music therapists who have experience/expertise in the use of technology) would be enriching to those who have less technology-related knowledge and experience, and would provide a platform for discussing the potential sociocultural constraints and hesitations in the use of technology. In addition, adopting the participatory approach of including the stakeholders (patients) themselves may create new and surprising insights about the use of technology in MT sessions, and how this use is related to their (digital) daily life.

## Data Availability Statement

The dataset presented in this article is not readily available due to compliance with NUS ethics board policies regarding data confidentiality. Requests to access the datasets should be directed to KA, katagres@nus.edu.sg.

## Ethics Statement

The studies involving human participants were reviewed and approved by National University of Singapore Institutional Review Board. The patients/participants provided their written informed consent to participate in this study.

## Author Contributions

KA led the project and manuscript writing, in collaboration with KF and SS. KF and KA led the participant recruitment. SS and KA created the data tables. KA performed the statistical analyses. KA and SS created the survey questions, with input from KF. All authors contributed to the data analyses and contributed meaningfully to the research study and manuscript preparation.

## Conflict of Interest

The authors declare that the research was conducted in the absence of any commercial or financial relationships that could be construed as a potential conflict of interest.

## Footnotes

1 Eligible music therapists were found by contacting both national-level and independently-owned music therapy organizations in Asia, Europe, and North America. The following music therapy organizations were contacted about taking part in the study: Association for Music Therapy (Singapore), American Music Therapy Association, New York Creative Arts Therapists (US), Canadian Association of Music Therapists, Music Therapy Association for Alberta (Canada), Music Therapy New Zealand, the Australian Music Therapy Association, Austrian Music Therapy Confederation (Austria), Association for (ethno) Music Therapy (Austria), Belgian Professional Association for Music Therapy (Belgium), Danish Association of Music Therapy (Denmark), Deutsche Musiktherapeutische Gesellschaft (Germany), Italian Professional Association of Music Therapy (Italy), Dutch Association of Music Therapy (Netherlands), Asociación Internacional de Musicoterapeutas (Spain), Asociación de Musicoterapia (Spain), Swiss Association for Music Therapy, European Music Therapy Confederation, and the World Federation of Music Therapy.
